# Experimental evolution reveals trade-offs between mating and immunity

**DOI:** 10.1098/rsbl.2013.0262

**Published:** 2013-08-23

**Authors:** Kathryn B. McNamara, Nina Wedell, Leigh W. Simmons

**Affiliations:** 1 Centre for Evolutionary Biology, The University of Western Australia, Crawley 6009, Australia; 2 Centre for Ecology and Conservation, University of Exeter, Cornwall Campus, Penryn TR10 9EZ, UK

**Keywords:** sexual selection, sperm competition, immune function

## Abstract

Immune system maintenance and upregulation is costly. Sexual selection intensity, which increases male investment into reproductive traits, is expected to create trade-offs with immune function. We assayed phenoloxidase (PO) and lytic activity of individuals from populations of the Indian meal moth, *Plodia interpunctella*, which had been evolving under different intensities of sexual selection. We found significant divergence among populations, with males from female-biased populations having lower PO activity than males from balanced sex ratio or male-biased populations. There was no divergence in anti-bacterial lytic activity. Our data suggest that it is the increased male mating demands in female-biased populations that trades-off against immunity, and not the increased investment in sperm transfer per mating that characterizes male-biased populations.

## 1. Introduction

The interplay between investment into reproduction and immunity is central to our understanding of sexual selection [1]. The maintenance and upregulation of immune function is costly, and resource limitation requires that it is traded-off against investment into both pre- and post-copulatory reproductive traits [2]. While there is a growing body of evidence for phenotypic trade-offs between immunity and post-copulatory reproductive traits, evolutionary changes in immunity in response to selection for varying reproductive investment is less well documented [3,4].

Experimental evolution studies have documented rapid evolutionary changes in male reproductive investment in response to increased sexual selection [5–7]. With increasing female mating frequency, the concomitant increase in observed male reproductive investment, such as testes size, is typically attributed to the effect of selection via sperm competition [6,7]. Yet, at a population level, under an equal sex ratio (ESR), increases in female mating frequency should be tightly associated with increases in male mating frequency [8]. Thus, the elevated male investment observed when female mating rate increases could potentially be driven by sperm competition to produce more sperm per ejaculate, or by the demand for more ejaculates. Separating out these two mechanisms is difficult (for a review, see Vahed & Parker [8]). Experimental evolution studies that manipulate adult sex-ratio bias can simultaneously alter the intensity of sexual selection and male mating rates. Observation of the nature of immune function resource trade-offs with these two traits can provide insight into the selection pressure promoting increased male reproductive investment.

We used an experimental evolution approach to explore genetic trade-offs between investment in reproduction and immune function in the polygamous Indian meal moth, *Plodia interpunctella*. Males exhibit phenotypic [9] and genetic changes in reproductive investment in response to increased sperm competition. Using experimental evolution lines, Ingleby *et al*. [10] documented evolutionary responses to variation in the adult sex ratio. Because of the sex bias, in male-biased lines, female mating frequency was higher, with sperm competition promoting increased sperm investment per mating by males in these populations. Yet, in female-biased lines, with no sperm competition, males mated more frequently. Thus, these populations allow dissection of the selection pressures promoting trade-offs between immunity and alternative mechanisms of increased male investment. Using these same populations, we explored differences in immune investment for both males and females. We measured two components of invertebrate immune function: the ability to lyse and kill pathogens *in vitro* (lytic activity) and haemolymph concentrations of the enzyme phenoloxidase (PO), which underlies the cascade that results in encapsulation of foreign bodies and correlates with an organism's ability to resist pathogens and parasites [11,12]. Our data show that these populations have diverged in male investment into immunity, but that this divergence is more likely to be driven by the costs of multiple mating for males in female-biased populations than the costs of increased sperm transfer in response to sperm competition in male-biased populations.

## 2. Material and methods

### (a) Experimental evolution

Moths were reared at the University of Exeter, Cornwall, UK, on a diet of bran, yeast, honey and glycerol, and were maintained at 28°C with a 16 L : 8 D cycle. Three adult sex-ratio treatments, each with three replicates, were established from the wild-caught stock population. The female-biased (1 : 3 male : females), equal (1 : 1 male : female) and male-biased (3 : 1 males : female) sex-ratio treatments were maintained at 120 adults at each generation for approximately 80 generations (see Ingleby *et al*. [10]). Importantly, sex-ratio bias was only enforced at the adult stage, and larvae were reared under identical conditions. This design provided a common garden environment to test for genetic changes in immune function. Only two of the three equal sex-ratio lines could be assayed.

### (b) Immune assays

Fifth-instar wandering larvae (a distinct developmental stage) were haphazardly selected and weighed from each of the eight replicates (testes are visible through the cuticle). To account for potential variation in immune traits due to development time, we noted the days elapsed from population establishment to sampling. Haemolymph was drawn from decapitated larvae for lytic assays (2 µl) and PO assays (2 µl haemolymph: 20 µl ice-cold PBS) and frozen at −80°C.

PO was measured using established protocols [13] for 20 males and females in each replicate (except in one female-biased replicate, males = 18, females = 19). For each sample, 10 µl of haemolymph was added to a 96-well plate with 100 µl of 5 mM dopamine hydrochloride (Sigma–Aldrich H8502) in duplicate. The plate was incubated for 5 min and the absorbance measured at 492 nm every minute for 20 min (at 28°C) using a M5 SpectraMax microplate reader (Molecular Devices, Sunnyvale, CA).

A lytic zone assay was used to determine the anti-bacterial response for 16 males and females in each replicate to the bacterium *Arthrobacter globiformis* (ATCC reference no. 8010). Agar plates were made with 5 ml of 1 per cent nutrient agar containing a 1.25 per cent final concentration of overnight culture (modified from Haine *et al*. [14]). For each sample, 1 µl of haemolymph was pipetted in replicate onto the plate. The plates were incubated at 28°C for 48 h. The zone cleared was calculated as the average of two measurements of the zone's diameter, taken at two points perpendicular to each other.

### (c) Statistics

Data were analysed using linear mixed-effect models fit by REML (JMP v. 9, 2000). Replicate was nested within mating-bias treatment as a random effect. Non-significant interactions were removed from final models. Sex-specific standardized weights were calculated to account for inherent sex-differences in weight. Values of 0 for lytic (*n* = 1) and PO (*n* = 3) raw data were excluded from analysis.

Data deposited in the Dryad repository: doi.org/10.5061/dryad.bs385.

## 3. Results

### (a) Lytic activity

Lytic activity was not affected by adult sex ratio (*F*_2,249_ = 0.09, *p* = 0.92), or the sex (*F*_1,249_ = 0.06, *p* = 0.80), or the developmental durations of the population (*F*_1,249_ = 0.23, *p* = 0.64). Lytic activity, however, was lower for heavier individuals (*F*_1,249_ = 4.07, *β* = −0.12 ± 0.06, *p* = 0.046). A non-significant interaction between mating bias and sex was removed from the model (*F*_2,247_ = 0.04, *p* = 0.96).

### (b) Phenoloxidase activity

**Figure 1.**
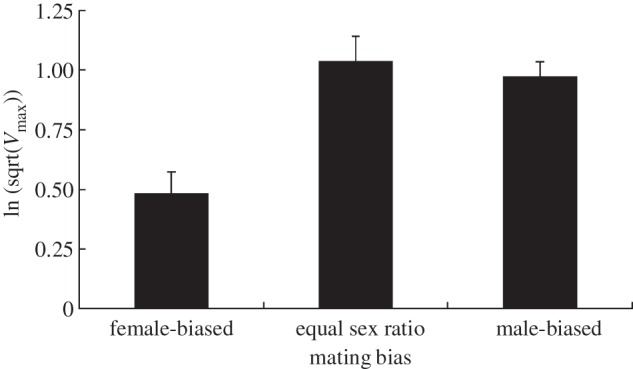
Least square mean (± s.e.) phenoloxidase activity (*V*_max_) among populations of *Plodia interpunctella* evolving under varying adult sex ratios.

PO was affected by sex-ratio treatment: individuals from female-biased populations had lower PO (*F*_2,308_ = 8.91, *p* = 0.03; figure 1). PO was lower in females (*F*_1,308_ = 4.16, *p* = 0.04) and in faster developing populations (*F*_1,308_ = 27.31, *β* = 0.09 ± 0.02, *p* = 0.003), while the relationship between PO and weight was not significant (*F*_1,308_ = 3.12, *β* = −0.09 ± 0.04, *p* = 0.08). A non-significant interaction between mating bias and sex was removed from the model (*F*_2,306_ = 0.04, *p* = 0.96).

## 4. Discussion

We found that males and females evolving under a female-biased adult sex ratio evolved to invest less in a key component of the innate immune system, PO. Two previous studies of evolutionary trade-offs between male reproductive investment and immunity found negative relationships between sexual selection intensity and immunity [3,4]. We observed the opposite pattern: individuals from male-biased populations in which sperm competition has selected for the transfer of greater numbers of sperm [6], had an immune function comparable to populations evolving under an even sex ratio where selection from sperm competition was weaker. In the female-biased lines, males mate three times more frequently than males in the male-biased lines [10]. Thus, although males in the male-biased treatments transfer more sperm per mating, male sperm expenditure is greater in female-biased lines. Consistent with phenotypic studies in other species [2], the greater sperm investment by males in the female-biased lines comes at a cost to investment in immunity, specifically PO. Thus, it is not the intensity of selection from sperm competition that drives an evolutionary reduction in male immune investment, but rather selection for increased male mating demands. Surprisingly, the divergence observed in mating traits between ESR and male-biased lines was not reflected in PO. ESR males mate approximately twice as frequently as male-biased lines, but transfer significantly fewer sperm, potentially creating a comparable male reproductive effort. A quantitative analysis of total reproductive expenditure, that includes testes size, sperm per ejaculate and spermatophore mass, is needed to elucidate these relationships.

How female immunological responses should change in response to variation in sexual selection is less clear. All things being equal, sexual dimorphism in immunity is predicted to increase with sexual selection intensity [15]. However, studies of the genetic architecture of immunity demonstrate positive genetic correlations between the sexes in measures of immunity [16], even when populations are subject to increased sexual selection intensity [4]. Such studies point to intralocus sexual conflict, with neither sex maintaining optimal immunity [16]. Intralocus sexual conflict and genetic correlations have been demonstrated in multiple fitness traits in *P. interpunctella* [17]. Our data show that the degree of sexual dimorphism in immune function did not diverge in response to selection, a result predicted by the genetic constraint model. In general, females had lower PO than males, consistent with previous work in this species [18], but in contradiction to classic models predicting stronger female immunity [19]. Such models are based on the traditional dichotomy between low male and high female reproductive investment. However, like all Lepidoptera, male *P. interpunctella* make a substantial reproductive investment at each mating—approximately 4 per cent of the male's body mass [20]. It may be that the magnitude of male investment in ejaculate provisions transferred to females is sufficient to alter the relative immune costs of reproduction incurred by males and females. While selection on male mating frequency is the most parsimonious explanation for the immunological changes observed, it should be noted that sex ratio manipulation may also affect other unmeasured traits, such as female physiology and behaviour, which may also affect female immune investment.

Only one component of the immune system traded-off against reproductive investment. Why PO is more labile than lytic activity in this species is not clear. PO can change plastically in this species in response to a range of factors, including larval density and diet [13]. Potentially, maintaining PO levels is also more costly; there is experimental evidence for the costs of selection for high PO levels in other species [21], while PO is a condition-dependent trait in *P. interpunctella* [13]. It is also unclear why PO, but not lytic, activity varies according to the developmental duration of each replicate, but this may be due to the time available for the acquisition of nutritional resources.

In conclusion, using immune function trade-offs, we show that it is male mating rate, and not sperm competition, that is the likely selection pressure for the evolution of decreased immune investment in this species. We demonstrate the importance of considering species-specific costs of ejaculate transfer when making predictions using traditional models of resource trade-offs. We also highlight the importance of considering female immune responses to sexual selection on males, as this phenomenon has the potential to reveal genetic constraints that might shape the architecture of immune system evolution.
